# Minimally Invasive Floating Metatarsal Osteotomy for Diabetic Foot Ulcers: A Systematic Review and Meta‐Analysis

**DOI:** 10.1111/wrr.70123

**Published:** 2026-01-10

**Authors:** Arthur Tarricone, Collin E. Pehde, Lee C. Rogers, Allen Gee, Karla De La Mata, Kimberly Barron, Ian Barron, Alexandria A. Armstrong, Robert Frykberg, Nitish Tirugnanasambandam, Lawrence A. Lavery

**Affiliations:** ^1^ Department of Orthopaedics University of Texas at San Antonio San Antonio Texas USA; ^2^ Department of Medicine, Mount Sinai Morningside and West Hospitals Icahn School of Medicine at Mount Sinai New York New York USA

## Abstract

Minimally invasive floating metatarsal osteotomy has been proposed as a surgical strategy to address recurrent or persistent diabetic foot ulcers (DFUs) by correcting underlying biomechanical deformities. We conducted a systematic review and meta‐analysis in accordance with PRISMA guidelines, querying four databases through September 2025 for studies involving adult patients with neuropathic DFUs treated with minimally invasive floating metatarsal osteotomy and followed for at least 12 months. Six studies comprising 184 subjects (176 with DFUs, 8 prophylactic) met inclusion criteria. Pooled outcomes demonstrated a healing rate of 98% (95% CI: 0.94–1.00) with a mean time to closure of 31.7 days (95% CI: 24.1–39.3). Ulcer recurrence occurred in 4% (95% CI: 0.02–0.09), while transfer lesions developed in 14% (95% CI: 0.08–0.20) and nonunion was observed in 14% (95% CI: 0.06–0.29). The overall infection rate was 7% (95% CI: 0.04–0.12). These findings indicate that minimally invasive floating metatarsal osteotomy is a safe and effective surgical option for off‐loading neuropathic DFUs; demonstrating high healing rates, rapid time to closure and low recurrence when compared with conservative care. Larger randomised controlled trials are warranted to validate these results and establish standardised surgical indications.

## 1 Introduction

Minimally invasive floating metatarsal osteotomy is a surgical procedure that can be used to treat or prevent recurrent or persistent foot ulcers located under the metatarsal heads in patients with diabetes‐related peripheral neuropathy. Patients with peripheral neuropathy and increased pressure on a plantar‐deviated metatarsal head frequently develop foot ulcers, which can lead to serious complications such as infection, hospitalisation, amputation and death [1, 2]. The annual incidence of foot ulcers in people with diabetes is about 2%–7%, and they are a causative factor in up to 84% of diabetic foot amputations [3, 4]. Approximately 25% of patients with diabetes will develop a diabetic foot ulcer (DFU) in their lifetime [5, 6].

Conservative treatments of diabetic ulcers, such as off‐loading with casts, boots, braces, special shoes and orthotics, are often effective for healing the initial ulceration [5, 7]. However, these methods can fail due to a lack of patient compliance or complications, and they do not correct the underlying anatomical deformity, leading to high recurrence rates. The recurrence rate for DFUs can be as high as 40% at 1 year and 70% at 3 years with conservative management alone [6, 8, 9]. When conservative treatment fails, surgical off‐loading may be indicated to decrease the pressure on the affected metatarsal head. The goal of surgery is not only to heal the current ulcer, but also to prevent recurrence by correcting the underlying foot deformity. The minimally invasive floating metatarsal osteotomy procedure is a technique that aims to reduce plantar pressure with minimal soft tissue damage and a low complication rate. This procedure was described over 50 years ago by Addante to treat intractable plantar keratoses [10].

The current minimally invasive osteotomy is also made at the surgical neck of the metatarsal to elevate the metatarsal head, which off‐loads the area and promotes ulcer healing. This approach has been shown to successfully reduce both peak plantar pressure and pressure time integral under the osteotomized metatarsal [11].

The purpose of this meta‐analysis is to evaluate the clinical results and complications of minimally invasive floating metatarsal osteotomy for isolated forefoot ulcerations to provide a comprehensive overview of its safety and effectiveness.

## 2 Methods

A literature search was conducted across four databases: PubMed/Embase, Medline and Cochrane central register of controlled trials. Medical subject (MeSH) and Boolean operators were employed in the final search was performed on 7 September 2025. Keywords included Floating Osteotomy, Mini‐invasive Floating Metatarsal Osteotomy, Minimally Invasive Metatarsal Osteotomy, Diabetes, Diabetic Foot and Diabetic Foot Ulcer. This meta‐analysis was conducted in accordance with the preferred reporting items for systematic reviews and meta‐analyses (PRISMA) checklist. This study protocol was registered in: Research Review Registry: 2049. Approval for this meta‐analysis did not require Institutional Review Board (IRB) approval due to the nature of the study design; however, local IRB approval was granted for all included studies.

After initial literature searches were completed, duplicates were removed. The remaining citations were screened by their titles and abstracts against the inclusion and exclusion criteria to determine relevance to the study topic. Included studies consisted of subjects treated with minimally invasive/floating osteotomies and followed for a minimum of 1 year.

Excluded studies included case reports, basic science research, non‐human populations, non‐English translatable studies, systematic reviews, meta‐analyses and subject populations with interventions above the knee. The screening process was conducted by two independent reviewers (L.A.L. and A.T.), with any disagreements resolved through third reviewer consensus (C.E.P.). A further reference list was also created to capture articles not found during initial database searches but relevant to the study. All categorical variables were reported as *n* (%) and continuous variables as ±standard deviations. Forest plots, Egger's test and funnel plots were generated using R Studio. All additional calculations were performed using StataBE17. For determining standard deviation from range, we utilized the standard formula of (range (max) – range (min))/4. A risk of bias assessment was determined using the Cochrane risk of bias tool 2. Heterogeneity was calculated using an *I* ^2^ and a random effects model was used in all analytics. A *p* value of 0.05 was considered significant.

## 3 Results

A total of 55 articles resulted from initial data query. After screening based on inclusion criteria, six studies were included in this meta‐analysis [11, 12, 13, 14, 15, 16]. In total, there were 184 subjects across the six studies, of which 146 were males. Out of the total subjects, 176 were treated for DFUs while 8 had prophylactic surgery. Patients' follow up time varied between studies, with the shortest average being 11.5 months and the longest at 26 months. All studies included in this analysis used a dorsal approach with a 3–5 mm incision and use of a Burr, with the exception of one study that discussed the use of a minimally invasive technique; however, it did not specify the incision size or use of a Burr [11, 12, 13, 14, 15, 16].

The average glycated haemoglobin across all studies ranged from 7.1 to 8.1. Three studies reported insulin use in subjects, *n* = 47. Extensive comorbidity data was reported in two studies and included categories such as smoking, cerebrovascular disease, congestive heart failure, chronic kidney disease, peripheral artery disease, retinopathy and nephropathy history. The remaining studies did not discuss the comorbidities described above (Table 1).

**TABLE 1:** Study demographics.

Study	Design	*n* total	Age^a^	Males	Ulcer grade	On insulin	HBA1C^a^	HTN	Smoking	Nephropathy	Retinopathy	CVA	CAD	PAD	Discussion of shoes
Boix 2024	Retrospective	25	59 [53, 66]	20	Unspecified	N/A	7.5 [6.7, 8.35]	16	14	11	14	1	3	12	No
Tamir 2016	Retrospective	17	58 [56, 63]	16	Texas Grade 1A	11	8.1 [6.5,10.5]	N/A	N/A	N/A	N/A	N/A	N/A	0	Yes
Tamir 2021	Retrospective	32	60.1 ± 8.5	29	Texas A0 in 3, A1 in 30 and A2 in 1 subject	22	7.9 ± 1.9	N/A	N/A	N/A	N/A	N/A	N/A	N/A	Yes
Tamir 2022	Retrospective	54	61 ± 9	46	Texas Grade 0A: 1, Grade 1A: 68, Grade 2A: 2	N/A	7.9% ± 1.9%	N/A	N/A	N/A	N/A	N/A	N/A	N/A	Yes
Biz 2017	Prospective	30	66.7 [53–75]	20	Texas 1A = 4; 2A = 2; 3A = 2; 2B = 6; 3B = 15; 2C = 2; 3C = 1; 3D = 3	14	7.13 ± 0.82	N/A	14	30	N/A	N/A	N/A	18	Yes
Mehlhorn 2020	Prospective	26	62 ± 9	15	Wagner ulcer unknown N	N/A	7.8 ± 0.1	N/A	N/A	N/A	N/A	N/A	N/A	N/A	Yes

^a^ Continuous variables reported as mean ± standard deviation or median [interquartile range].

Ulcers were graded by the University of Texas Ulcer Classification system in four studies [17]. The most common ulcer grade in this meta‐analysis was Grade 1A. One study used the Wagner classification (Table 1).

**FIGURE 1:**
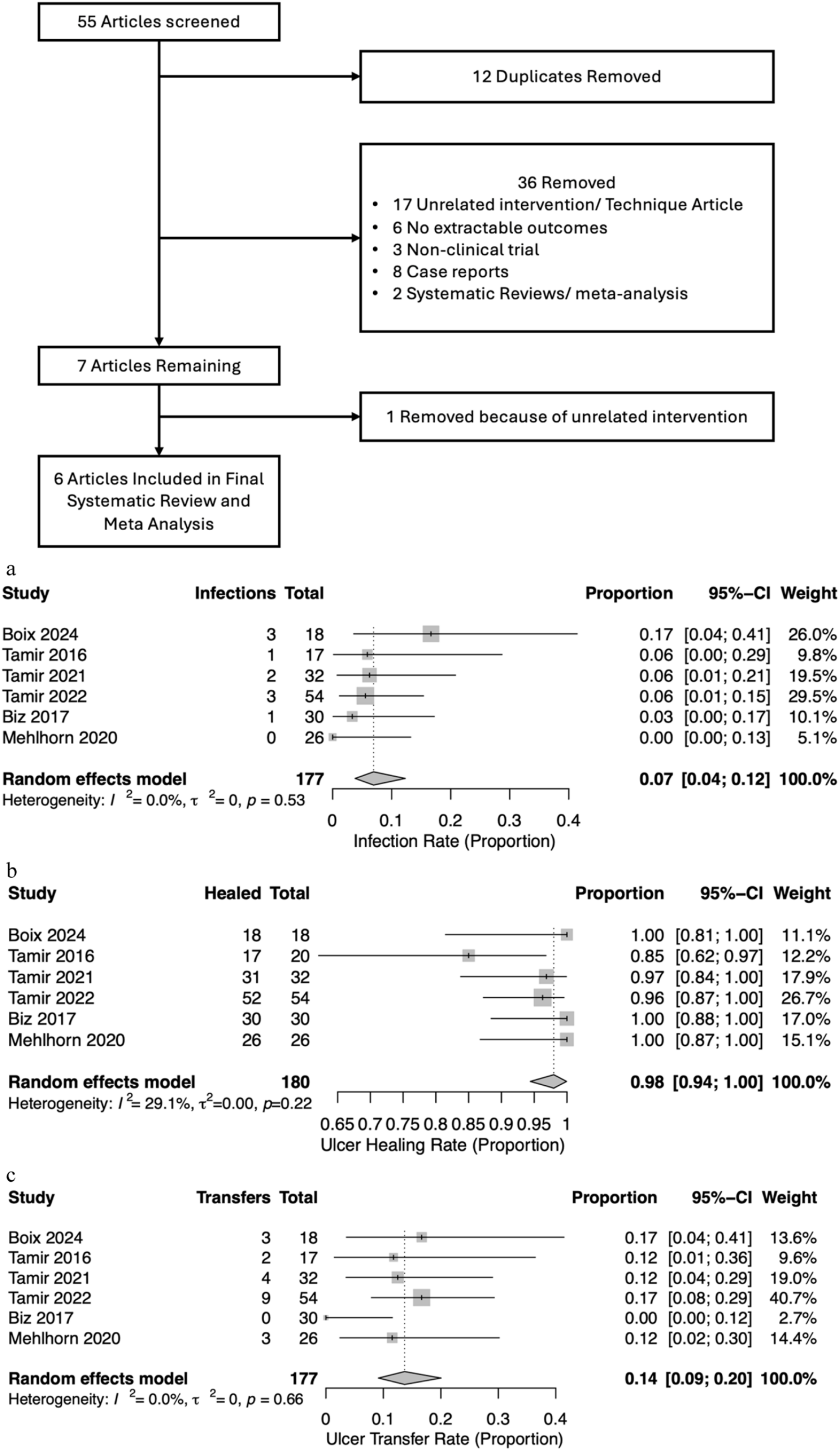
Flowchart of study inclusion. (a) Infections. (b) Ulcer healing. (c) Ulcer transfer.

**FIGURE 2:**
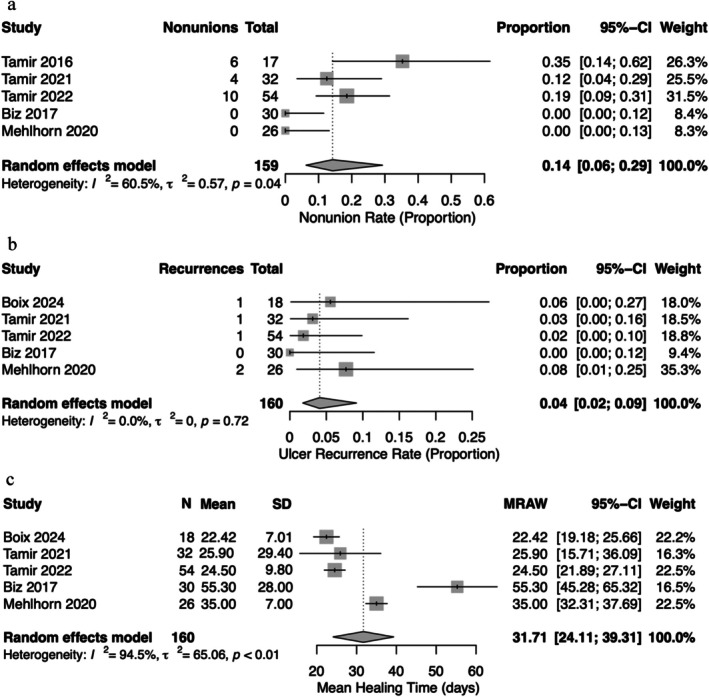
(a) Nonunion. (b) Ulcer recurrence. (c) Time until healing.

The primary outcomes assessed included infection, ulcer healing, ulcer transfer (Figure 1), time until healing, nonunion and ulcer recurrence (Figure 2). Infections were reported in all studies with a total of nine events, and the summative event proportion was 7% (0.04, 0.12) across all studies. Likewise, ulcer healing was reported in all studies with an event proportion of 98% (0.94, 1.00). Ulcer transfer was reported in all studies, with an event proportion of 14% (0.08, 0.20).

Nonunion and recurrence were only reported in three studies [11, 13, 14]. There was a total of 20 nonunion events, with a summative event proportion of 14% (0.06, 0.29). A total of five ulcers recurred, with an event proportion of 4% (0.02, 0.09). While all studies reported time to ulcer healing, one study only reported an average of 7 days without standard deviation. Therefore, that study was excluded from the final analysis of time to ulcer healing. The average time until healing for ulcers varied significantly between studies, with an overall 31.71 (24.11, 39.31) days.

## 4 Discussion

The present meta‐analysis demonstrates that minimally invasive floating metatarsal osteotomy is a highly effective surgical procedure for the off‐loading and healing of DFUs with a low complication rate. The combined event proportion for ulcer healing was 0.98 (0.95, 1.00), with an average healing time of 32.83 days. This healing rate is particularly significant when compared to the prolonged and often unsuccessful outcomes associated with conservative therapies alone. While off‐loading with total contact casts and other orthotics is the standard of care (SOC) for initial ulcer management, these methods are frequently challenged by patient compliance issues and do not address the underlying biomechanical deformity [7].

The persistent pathophysiological risk factors—including peripheral neuropathy, foot deformity and compromised perfusion—that lead to DFUs are not resolved by conservative wound healing, resulting in a recurrence rate that can reach 70% within 3 years and highlighting the necessity of a more comprehensive and durable therapeutic strategy [6, 8, 9, 10]. The results of this study found a pooled ulcer recurrence event proportion of only 4% (0.02, 0.09) for the minimally invasive floating metatarsal osteotomy procedure. This reduction in recurrence risk highlights the procedure's efficacy in addressing the root cause of the ulceration: excessive plantar pressure on a prominent metatarsal head.

A critical consideration in any surgical off‐loading procedure is the potential for infection as a complication of the procedure. The overall infection rate was low at 7% (0.04, 0.12) with nonunion occurring at a pooled event proportion of 14% (0.06–0.29). In addition, there was a low rate of new ulcers forming under adjacent metatarsal heads (transfer lesions). The pooled event proportion for ulcer transfer in our analysis was 14% (0.09, 0.20). This is a notable finding, as transfer lesions are a well‐documented risk with other metatarsal shortening osteotomies and procedures that resect the metatarsal head [18, 19]. This low complication profile further supports the safety and viability of the minimally invasive approach. In contrast, the results of a meta‐analysis of the SOC arm of RTCs to treat DFUs reported a pooled healing incidence of 33.2%, infection incidence of 17.4% and an average healing time of 50.1 days [20].

Our findings support the role that minimally invasive floating metatarsal osteotomies not only heal the current ulceration but reduce the risk of re‐ulceration. Surgical interventions have consistently proven to be an effective approach for treating neuropathic DFUs. A randomised controlled trial on non‐infected neuropathic DFUs found that surgery was more effective than conventional treatment, achieving a 95.5% healing rate versus 79.2% and significantly reducing healing time from 128.9 days to just 46.7 days, while also leading to fewer complications [21]. For distal toe wounds caused by flexible hammertoes, a recent meta‐analysis of 11 papers affirmed the efficacy of flexor tenotomies, showing healing rates from 92% to 100% and a rapid mean healing time of 2–4 weeks [22]. Similarly, a retrospective study on isolated plantar metatarsal DFUs found metatarsal head resection (MHR) surgery to be superior to medical management, with a 100% wound healing rate compared to 60%. The surgical group also experienced significantly faster healing (373.4 vs. 384.1 days). In addition, there was no ulcer recurrence compared to 16% recurrence in the medical group [23]. For neuropathic forefoot wounds stemming from dislocated metatarsophalangeal joints, pan‐metatarsal head resection has been found to reduce healing time to 60.1 days, decrease infection rates (35.5% vs. 64.5%) and lower ulcer reoccurrence (15.2% vs. 39.1%) compared to SOC [24]. Finally, for plantar hallux ulcerations secondary to hallux rigidus, first metatarsophalangeal joint resectional arthroplasty (Keller procedure) has been shown to heal wounds significantly faster (24.2 vs. 67.1 days) and result in fewer recurrent ulcers (4.8% vs. 35.0%) while maintaining comparable rates of infection and amputation [25].

This study's findings evaluating floating metatarsal osteotomies are consistent with existing literature on similar procedures, such as MHR, which has also shown high healing rates for chronic plantar ulcers [26, 27]. However, floating metatarsal osteotomies may offer advantages over head resections by preserving the metatarsal head and articular cartilage, theoretically maintaining a more stable bony architecture. Additionally, the minimally invasive nature of the procedure leads to less soft tissue trauma, which is particularly crucial in a diabetic population with impaired wound healing. Finally, minimally invasive floating metatarsal osteotomies can play a role in lifestyle benefit. This includes earlier return to wearing shoes, improved independence including walking and driving and less time confined to a cast or walker [28]. In addition, by correcting the underlying biomechanical dysfunction, this may reduce ulcer recurrence rates [11, 12, 13, 14, 15, 16].

Limitations of this meta‐analysis include the small number of studies and the variability in reported outcomes and follow‐up times. Only a subset of studies reported nonunion and ulcer recurrence, and the data on comorbidities was inconsistent. Future studies should standardise reporting to allow for more robust meta‐analyses. Specifically, a larger number of patients and longer follow‐up periods are needed to confirm the long‐term outcomes to better evaluate the true incidence of complications like nonunion and transfer lesions. High‐quality, randomised controlled trials comparing minimally invasive floating osteotomy to other surgical and conservative off‐loading techniques are needed to establish a definitive treatment algorithm for these challenging clinical cases.

Table 1 describes the general study characteristics for all included citations. Ulcer grading was reported according to the Texas or Wagner classifications across all studies.

Table 2 reports the clinical outcomes for each study included in this meta‐analysis. Outcomes include ulcer healing, healing time (days), ulcer recurrence, ulcer transfer, infection and nonunion.

**TABLE 2:** Clinical outcomes by study.

Study	*n* total	Ulcer healing rate	Healing time	Ulcer recurrence	Ulcer transfer	Infection	Nonunion
Boix 2024	25	18	22.42 ± 7.01 days	1	3	3	N/A
Tamir 2016	17	17	7 days	N/A	2	1	6
Tamir 2021	32	31	3.7 ± 4.2 weeks	1	4	2	4
Tamir 2022	54	52	3.5 ± 1.4 weeks	1	9	3	10
Biz 2017	30	30	7.9 ± 4 weeks	0	0	1	0
Mehlhorn 2020	26	26	5 ± 1 weeks	2	3	0	0

Figure 1 illustrates the forest plots for summative average for the primary outcomes of infections, ulcer healing and ulcer transfer. The reported findings do not omit any data from any of the studies included. All models utilized a random effects model, and heterogeneity was absent in the final analysis.

Figure 2 illustrates the forest plots for summative average for the primary outcomes of nonunion, ulcer recurrence and time until healing. Boix was excluded from nonunion, and Tamir et al. [12] was excluded from ulcer recurrence and time until healing. All models utilised a random effects model. Heterogeneity was present in nonunion and time until healing figures.

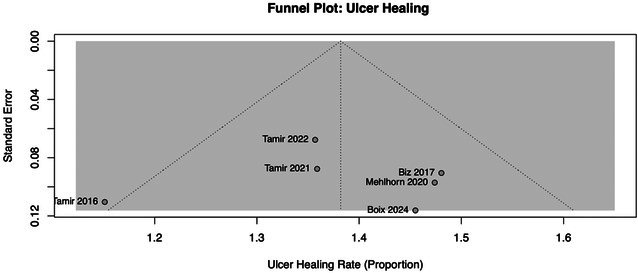

Eggers test

Test result: *t* = −0.22, df = 5, *p* = 0.8319.

Bias estimate: −0.5765 (SE = 2.5783).

## 5 Search Strategy

Pubmed/Medline

*Search*: (Floating Osteotomy OR Mini‐invasive Floating Metatarsal Osteotomy OR Minimally Invasive Metatarsal Osteotomy) AND Diabetes

(((“float”[All Fields] OR “floated”[All Fields] OR “floating”[All Fields] OR “floats”[All Fields]) AND (“osteotomie”[All Fields] OR “osteotomied”[All Fields] OR “osteotomy”[MeSH Terms] OR “osteotomy”[All Fields] OR “osteotomies”[All Fields])) OR (“Mini‐invasive”[All Fields] AND (“float”[All Fields] OR “floated”[All Fields] OR “floating”[All Fields] OR “floats”[All Fields]) AND (“metatarsal bones”[MeSH Terms] OR (“metatarsal”[All Fields] AND “bones”[All Fields]) OR “metatarsal bones”[All Fields] OR “metatarsal”[All Fields] OR “metatarsals”[All Fields]) AND (“osteotomie”[All Fields] OR “osteotomied”[All Fields] OR “osteotomy”[MeSH Terms] OR “osteotomy”[All Fields] OR “osteotomies”[All Fields])) OR (“Minimally”[All Fields] AND (“invasibility”[All Fields] OR “invasible”[All Fields] OR “invasion”[All Fields] OR “invasions”[All Fields] OR “invasive”[All Fields] OR “invasively”[All Fields] OR “invasiveness”[All Fields] OR “invasives”[All Fields] OR “invasivity”[All Fields]) AND (“metatarsal bones”[MeSH Terms] OR (“metatarsal”[All Fields] AND “bones”[All Fields]) OR “metatarsal bones”[All Fields] OR “metatarsal”[All Fields] OR “metatarsals”[All Fields]) AND (“osteotomie”[All Fields] OR “osteotomied”[All Fields] OR “osteotomy”[MeSH Terms] OR “osteotomy”[All Fields] OR “osteotomies”[All Fields]))) AND (“diabete”[All Fields] OR “diabetes mellitus”[MeSH Terms] OR (“diabetes”[All Fields] AND “mellitus”[All Fields]) OR “diabetes mellitus”[All Fields] OR “diabetes”[All Fields] OR “diabetes insipidus”[MeSH Terms] OR (“diabetes”[All Fields] AND “insipidus”[All Fields]) OR “diabetes insipidus”[All Fields] OR “diabetic”[All Fields] OR “diabetics”[All Fields] OR “diabets”[All Fields])

Translations

*Floating*: “float”[All Fields] OR “floated”[All Fields] OR “floating”[All Fields] OR “floats”[All Fields]

*Osteotomy*: “osteotomie”[All Fields] OR “osteotomied”[All Fields] OR “osteotomy”[MeSH Terms] OR “osteotomy”[All Fields] OR “osteotomies”[All Fields]

*Floating*: “float”[All Fields] OR “floated”[All Fields] OR “floating”[All Fields] OR “floats”[All Fields]

*Metatarsal*: “metatarsal bones”[MeSH Terms] OR (“metatarsal”[All Fields] AND “bones”[All Fields]) OR “metatarsal bones”[All Fields] OR “metatarsal”[All Fields] OR “metatarsals”[All Fields]

*Osteotomy*: “osteotomie”[All Fields] OR “osteotomied”[All Fields] OR “osteotomy”[MeSH Terms] OR “osteotomy”[All Fields] OR “osteotomies”[All Fields]

*Invasive*: “invasibility”[All Fields] OR “invasible”[All Fields] OR “invasion”[All Fields] OR “invasions”[All Fields] OR “invasive”[All Fields] OR “invasively”[All Fields] OR “invasiveness”[All Fields] OR “invasives”[All Fields] OR “invasivity”[All Fields]

*Metatarsal*: “metatarsal bones”[MeSH Terms] OR (“metatarsal”[All Fields] AND “bones”[All Fields]) OR “metatarsal bones”[All Fields] OR “metatarsal”[All Fields] OR “metatarsals”[All Fields].

*Osteotomy*: “osteotomie”[All Fields] OR “osteotomied”[All Fields] OR “osteotomy”[MeSH Terms] OR “osteotomy”[All Fields] OR “osteotomies”[All Fields]

*Diabetes*: “diabete”[All Fields] OR “diabetes mellitus”[MeSH Terms] OR (“diabetes”[All Fields] AND “mellitus”[All Fields]) OR “diabetes mellitus”[All Fields] OR “diabetes”[All Fields] OR “diabetes insipidus”[MeSH Terms] OR (“diabetes”[All Fields] AND “insipidus”[All Fields]) OR “diabetes insipidus”[All Fields] OR “diabetic”[All Fields] OR “diabetics”[All Fields] OR “diabets”[All Fields]

## 6 Results 16

*Search*: Floating Osteotomy AND Diabetes

(“float”[All Fields] OR “floated”[All Fields] OR “floating”[All Fields] OR “floats”[All Fields]) AND (“osteotomie”[All Fields] OR “osteotomied”[All Fields] OR “osteotomy”[MeSH Terms] OR “osteotomy”[All Fields] OR “osteotomies”[All Fields]) AND (“diabete”[All Fields]) OR “diabetes mellitus”[MeSH Terms] OR (“diabetes”[All Fields] AND “mellitus”[All Fields]) OR “diabetes mellitus”[All Fields] OR “diabetes”[All Fields] OR “diabetes insipidus”[MeSH Terms] OR (“diabetes”[All Fields] AND “insipidus”[All Fields]) OR “diabetes insipidus”[All Fields] OR “diabetic”[All Fields] OR “diabetics”[All Fields] OR “diabets”[All Fields]

Translations

*Floating*: “float”[All Fields] OR “floated”[All Fields] OR “floating”[All Fields] OR “floats”[All Fields]

*Osteotomy*: “osteotomie”[All Fields] OR “osteotomied”[All Fields] OR “osteotomy”[MeSH Terms] OR “osteotomy”[All Fields] OR “osteotomies”[All Fields]

*Diabetes*: “diabete”[All Fields] OR “diabetes mellitus”[MeSH Terms] OR (“diabetes”[All Fields] AND “mellitus”[All Fields]) OR “diabetes mellitus”[All Fields] OR “diabetes”[All Fields] OR “diabetes insipidus”[MeSH Terms] OR (“diabetes”[All Fields] AND “insipidus”[All Fields]) OR “diabetes insipidus”[All Fields] OR “diabetic”[All Fields] OR “diabetics”[All Fields] OR “diabets”[All Fields]

## 7 Results 5

Embase:

**Table:** 

Search number	Query	Results
1	Diabetic foot	24,373
2	Diabetic foot ulcer	2612
3	Minimally invasive metatarsal Osteotomy	1325
4	Distal minimally invasive Metatarsal osteotomy	12
5	1 and 3	31
6	2 and 3	3

Cochrane: None.

Risk of bias

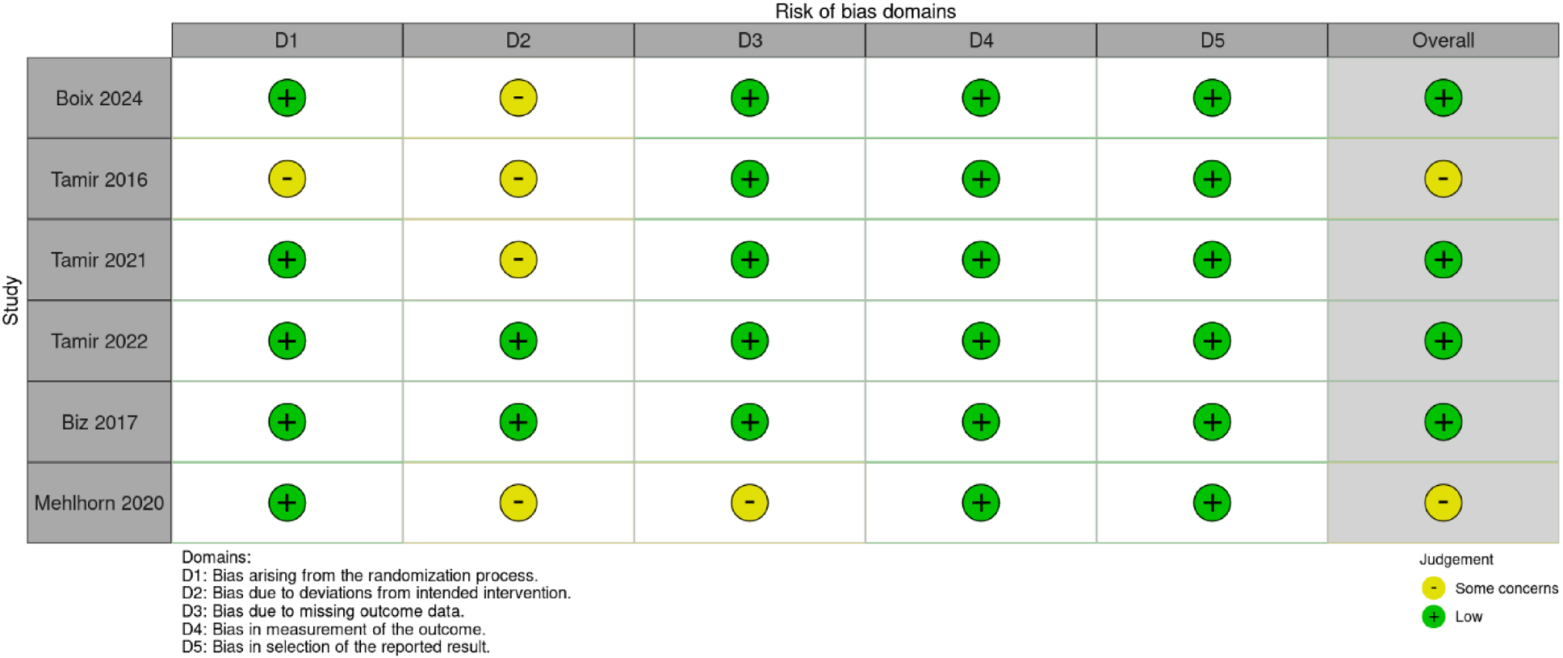

## Funding

The authors have nothing to report.

## Conflicts of Interest

The authors declare no conflicts of interest.

## Data Availability

The data that support the findings of this study are openly available in https://pubmed.ncbi.nlm.nih.gov/.
